# Glioma in the third trimester of pregnancy: Two cases and a review of the literature

**DOI:** 10.3892/ol.2013.1106

**Published:** 2013-01-04

**Authors:** JIE WU, YAN-HUI MA, TIAN-LONG WANG

**Affiliations:** Department of Anesthesiology, Xuanwu Hospital, Capital Medical University, Beijing 100053, P.R. China

**Keywords:** brain tumors, pregnancy, treatment, outcome research

## Abstract

We present two cases of glioma (WHO grade III) in pregnant females presenting in the third trimester. Gliomas during pregnancy are rare. At present, the association between gliomas and pregnancy is poorly understood and little has been reported with regard to the management of patients with gliomas in pregnancy. Management of these cases presents a medical dilemma. Gliomas during pregnancy pose a risk to maternal and fetal life. The benefit-to-risk ratio should be carefully evaluated and discussed prior to surgery. In the present cases, caesarean section (CS) followed by craniotomy was performed under the same general anesthesia at 34 weeks’ gestation. The mothers received radiotherapy and chemotherapy following surgery. They have been followed up to the present date and remain in good health. These two cases indicate that early CS followed by craniotomy is an effective choice in pregnant patients with gliomas at ≥34 weeks’ gestation. In the present study, we describe these two cases and review the literature with regard to gliomas during pregnancy.

## Introduction

Although maternal mortality due to obstetric causes has declined, there has been a relative increase in non-obstetric causes of maternal mortality and morbidity (1). Central nervous system diseases, including intracranial tumors, particularly malignant brain tumors [odds ratio (OR), 143] (2) and trauma, remain a leading cause of indirect maternal mortality. Primary central nervous system tumors occur in ∼6 in 100,000 females (3), but are rare during pregnancy. A study reported the incidence of maternal malignant brain tumors at 3.6 per 1 million live births (4). The management of women with malignant brain tumors during pregnancy has not been well evaluated.

The present study reports two cases of pregnant women in the third trimester with gliomas. The patients underwent caesarean section (CS) followed by craniotomy for brain tumor resection under the same general anesthesia at 34 weeks’ gestation and were managed successfully. At present, the management of gliomas has been widely established in the literature, but those occurring in pregnancy have been poorly discussed. The present study discusses the management of the two cases with a review of the literature.

## Case report

### Case 1

**Figure 1:**
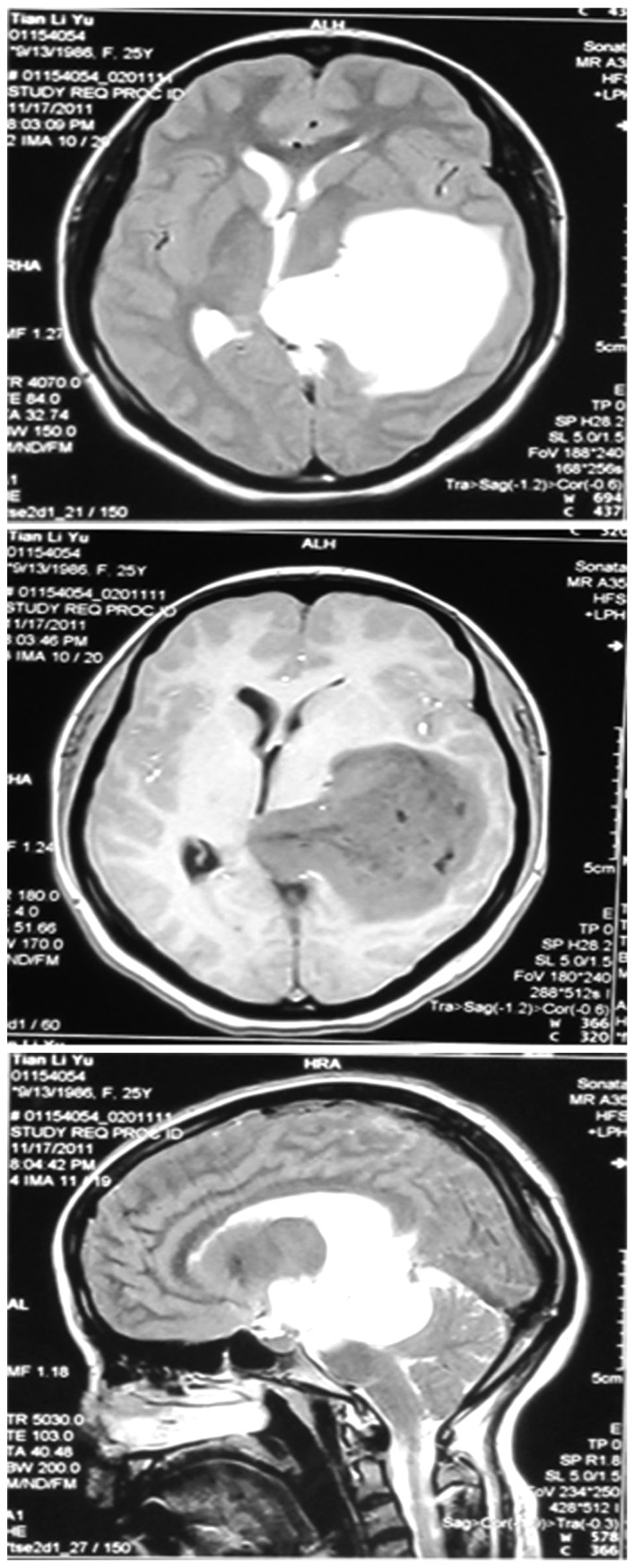
Images from a magnetic resonance imaging (MRI) scan of case 1.

A 25-year-old primipara patient was admitted to hospital at 32 weeks of pregnancy due to a headache for 1 month and blurred vision for 20 days. The patient was in the third trimester of pregnancy. Ultrasonography (USG) showed a single intrauterine live pregnancy with occiput presentation. A magnetic resonance imaging (MRI) scan showed a left frontoparietal mass (size: 6x5x5 cm) with peripheral enhancement (Fig. 1), with a shift of the median line.

After multidisciplinary consultation with the neurosurgery, obstetrics and anesthesiology departments, the initial decision was to delay the neurosurgery until after delivery. The patient and her family agreed with this strategy. The decision was made to treat the patient with dexamethasone to control the cerebral edema and accelerate fetal lung maturity. However, the patient’s visual acuity rapidly worsened. Therefore, the patient was scheduled for a CS and neurosurgical resection of the tumor under general anesthesia at 34 weeks’ gestation.

A live 1,900-g female was delivered 6 min after the skin incision. The neonate’s Apgar score at 1 min was 8 and after 2 min, the Apgar score sharply decreased to 3. The neonate was resuscitated immediately with intermittent positive pressure breathing and 0.04 mg naloxone was administered intramuscularly 5 min later. The Apgar score returned 8. The neonate was then admitted to the neonatal intensive care unit.

Oxytocin (10 units) was administered i.v. to improve the uterine contraction. Following delivery, the neurosurgical resection of the tumor was performed uneventfully. The patient recovered quickly from anesthesia without any neurological deficit and was extubated in the operating room.

The pathology laboratory revealed an astrocytoma, which was WHO grade III. The positive expression of glial fibrillary acidic protein (GFAP), CD34, p53, Olig-2, vimentin and isocitrate dehydrogenase-1 (IDH-1) were observed by immunohistochemical staining techniques. The patient was discharged 7 days after surgery. Adjuvant therapy was initiated 2 weeks after surgery. The patient completed radiotherapy and chemotherapy by taking temozolomide for 6 months post-craniectomy. The mother remained in a good condition and the baby was observed to develop normally throughout the 12-month follow-up period.

### Case 2

**Figure 2:**
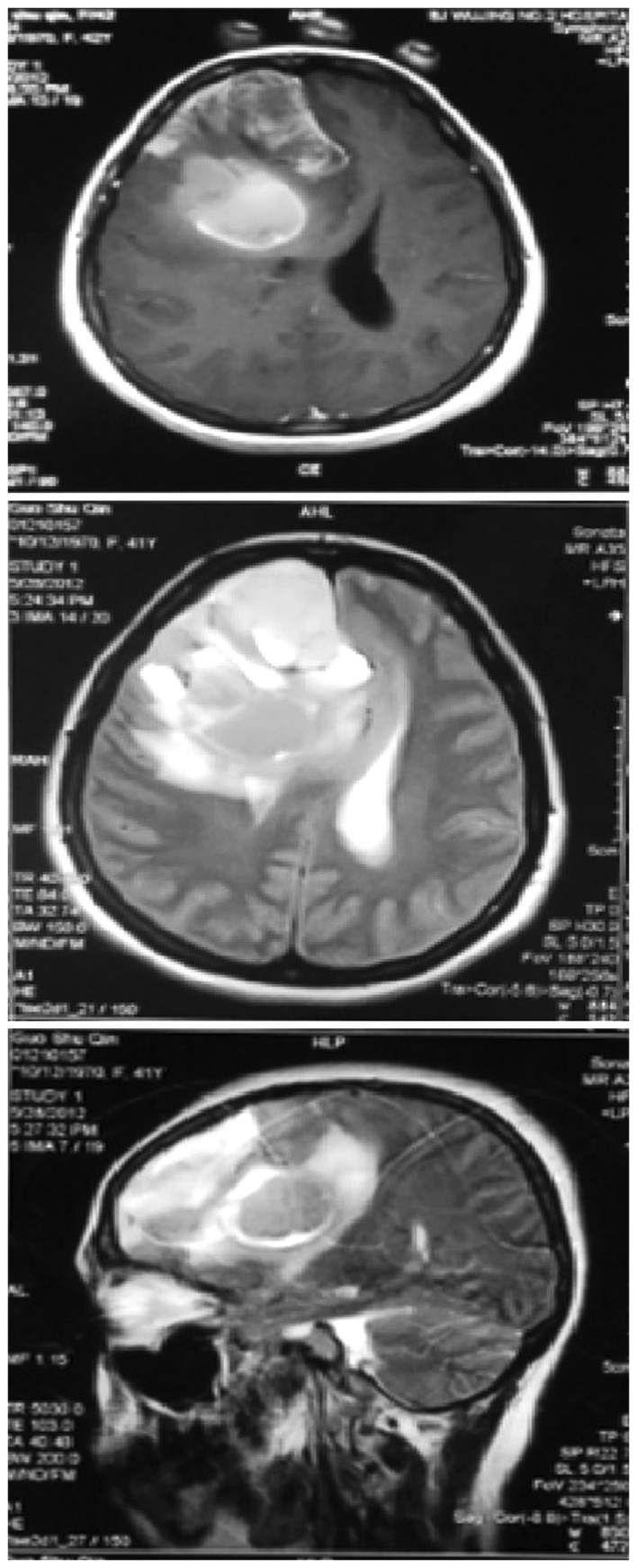
Images from a magnetic resonance imaging (MRI) scan of case 2.

A 42-year-old, gravida 4, para 3, patient was hospitalized at 34 weeks’ gestation due to a severe headache and vomiting for 15 days. The patient was admitted to the Xuanwu Hospital and was hemodynamically stable, with bilaterally reactive pupils. The patient’s previous obstetric history included three full-term pregnancies with two vaginal births and one CS. A physical examination confirmed weakness in the faculty of memory, indifference of facial expression and a Glasgow Coma Scale score of 13/15. An MRI scan showed a right frontal lobe tumor with an intratumoral hemorrhage (Fig. 2), causing a significant midline and brain shift toward the left side. Obstetric USG showed a single intrauterine live pregnancy with breech presentation which was concordant with its gestational age. The patient was otherwise healthy and denied any previous history of disease. The laboratory results were within the normal ranges. Due to the presence of an intratumoral hemorrhage, termination of the pregnancy was recommended. Following mannitol and steroid infusion, the patient was taken to the operation room for CS followed by neurosurgical resection of the tumor.

CS and craniotomy in the supine position were performed under general anesthesia. A healthy 2,450-g female was delivered by CS 5 min after the skin incision. The female neonate had an Apgar score of 9 at 1 min and 10 at 5 min after delivery and was taken to the neonatal intensive care unit for observation.

Ten units of oxytocin were administered intravenously to improve the uterine contraction after clipping the umbilical cord. The obstetrician massaged the uterus regularly to improve uterine tone during the neurosurgical procedure, which was performed uneventfully. At the completion of craniotomy for resection of the brain tumor, the patient was extubated while fully awake in the operating room and then transported to the intensive care unit.

The pathology laboratory reported an anaplastic oligodendroglioma, WHO grade III. Immunohistochemistry of the tumor cells revealed positive p53, IDH-1 and Ki-67 (40%) expression and partially positive GFAP, CD34 and EMA expression. The patient was discharged 8 days after surgery. The patient received radiotherapy 25 times and treatment with chemotherapy using temozolomide was administered in another hospital.

At follow-up 6 months after discharge, the mother was observed to have an adequate condition and the newborn was observed to be in a good neurological and behavioral state of development.

## Discussion

Intracranial tumors are extremely rare in pregnancy and the prevalence in pregnancy is unknown. Several types of brain tumors are associated with pregnancy, including meningiomas, gliomas, pituitary adenomas and acoustic neurinomas. Although a few studies have reported cases of women with gliomas during pregnancy, this situation remains exceptional. A French study on gliomas reported eight cases between 1992 and 2007; five patients had gliomas prior to pregnancy and three developed gliomas during pregnancy and diffuse gliomas were diagnosed (5). Ducray *et al*(6) reported four cases of gliomas, Lynch *et al*(7) five cases and Cutura and Soldo (8) one case. The present study reports two cases of glioma in the third trimester.

Intracranial hypertension usually occurs secondary to malignant brain tumors. The most common clinical manifestations of intracranial hypertension are headaches and vomiting, which were observed in the present patients. Intracranial hypertension may be misdiagnosed due to confusion with pregnancy. Headaches also occur in pregnancy, but a persistent headache is particularly associated with hyperemesis or neurological deficits in patients with brain tumors. In the two present cases, the patients complained of headaches, which occurred several weeks prior to hospital admission. A detailed history and MRI are likely to aid diagnosis.

Management of these cases presents a medical dilemma. A multidisciplinary group, including a neurosurgeon, obstetrician, anesthesiologist and neonatologist should evaluate whether the mother’s and the fetus’s lives are threatened. A multidisciplinary team recommends the optimal timing for the termination of pregnancy, as determined by the fetus maturity and mother’s neurological condition. The various treatment modalities in pregnant women with glioma are associated with histology and gestational age (6). The two present cases exhibited neurological deterioration or signs of severe intracranial hypertension. If the condition had progressed it may have threatened the mothers’ lives. Therefore, timely interventions were important for the two patients.

Neurosurgical procedures remain the major therapy for gliomas. Most craniotomy procedures are performed during pregnancy and after delivery in stable patients (5,9,10). Since the maternal intravascular volume increases with advancing gestational age, tumor resection is at risk of causing significant hemorrhaging. If the lesion is not large and the mother does not have intracranial hypertension, the tumor may be removed postpartum or its removal may be deferred until the fetus comes to term. However, if the prognosis of maternal survival is poor, craniotomy may be immediately performed (11,12). In the present cases exhibiting neurological deterioration, we considered that postoperative premature labor and fetus distress, which may lead to fetal deaths (13), were likely. In view of the condition of the two pregnant patients at 34 weeks’ gestation, with the aim of reducing morbidity and mortality (11), the decision was made to perform CS and craniotomy under the same anesthesia following the evaluation of fetal viability by an obstetrician. Since synthetic oxytocin has been used in patients with intracranial tumors without any adverse effects (3), oxytocin and regular uterine massage, which enhance uterine contraction, were administered to the patients to reduce postpartum hemorrhaging during the subsequent neurosurgery.

Adjuvant therapies, including radiotherapy and chemotherapy, appear to be effective for patients with gliomas. Early radiotherapy and chemotherapy significantly improves the quality of life of the patients. However, radiotherapy during pregnancy may cause harm to the developing fetus. Although the threshold dose of 0.1–0.2 Gy is low and is not sufficient for curative radiotherapy during pregnancy, radiation effects may appear (14). All chemotherapy drugs are capable of crossing the placenta and may induce fetal toxicity, particularly in the first trimester. In general, pregnant women with gliomas are advised to delay radiotherapy and chemotherapy until after delivery. If the CS is performed earlier, this allows more time to undergo further adjuvant treatment. In the present patients, CS followed by craniotomy was performed at 34 weeks’ gestation. The patients started early radiotherapy and chemotherapy with temozolomide, according to the regimen that is currently considered to be the standard care for glioblastomas (15).

Delivery by CS followed by neurosurgery is almost always performed under general anesthesia. Studies have shown that general anesthesia is safe and well tolerated for neuro-anesthesia during pregnancy (16). Airway management must address the avoidance of increases in intracranial pressure, the presence of a potentially full stomach, pregnancy-induced changes to the airway and enlarged breasts, which increase the incidence of a difficult intubation (12). It is important to preserve cerebral and uteroplacental perfusion by maintaining hemodynamic stability. Extubation should be delayed until the patient is sufficiently awake for the patient’s airway to be protected from regurgitation and pulmonary aspiration. The postoperative management of pregnant patients following neurosurgical intervention is similar to that of non-pregnant patients.

Malignant brain tumors are associated with adverse outcomes in pregnancy (2). Tewari *et al*(17) reviewed a case series of pregnancy-associated malignant brain tumors for two decades between 1978 and 1998 at five hospitals. This review reported that six out of eight patients delivered under emergency conditions. Of these six mothers, four died and two had significant neurological deficits. However, the outcomes for the women and their neonates reported in the present study were generally good.

Gliomas in patients during pregnancy are rare. The aim of treatment is to minimize maternal and fetal mortality and morbidity. A multidisciplinary approach, including obstetricians, anesthesiologists, neurosurgeons and neonatologists, is essential to achieve the optimal timing of neurosurgery and delivery. The findings from the present cases suggest that CS and craniotomy may be performed under the same general anesthesia at 34 weeks’ gestation. Further adjuvant treatment started soon after surgery may facilitate improved outcomes for the mother and newborn.
